# Levosimendan use in the emergency management of decompensated peripartum cardiomyopathy

**DOI:** 10.4103/0974-2700.58651

**Published:** 2010

**Authors:** Alina Uriarte-Rodríguez, Luciano Santana-Cabrera, Manuel Sánchez-Palacios

**Affiliations:** Universitary Hospital Insular in Gran Canaria, Las Palmas de Gran Canaria, Spain

Sir,

On the occasion of the recent publication about the interesting case report of postpartum cardiomyopathy (PPCM)[1], we would like to share our experience of a case refractory to the habitual treatment used in acute heart failure (AHF), which was treated with levosimendan obtaining a good clinical result, considering that the mortality rate of this disease is around 30–60%.

A 42-year-old woman, with medical treatment for hyperthyroidism, eutopic delivery a week before, and gestoses in the last 2 weeks of gestation, turned up in the emergency department of our hospital referring progressive dyspnea that ended up in dyspnea being at rest accompanied with orthopnea and paroxysmal nocturnal dyspnea.

On arrival in the emergency department, she was conscious, her blood pressure was 160/100 mmHg, and she presented tachycardia and taquipnea. Chest radiograph showed an image of acute pulmonary edema. Echocardiography revealed severe left ventricular dysfunction (LVEF 20%), normal left ventricular end diastolic dimension, and mild mitral regurgitation. In spite of the conventional treatment for AHF with diuretics and vasodilators, the patient did not improve, and was given mechanical ventilation and vasoactive drugs because of hypotension.

In this critical situation, she was transferred to the intensive care unit. In the presence of AHF refractory to the habitual treatment, administration of a continuos levosimendan infusion at 0.1 μg/kg/min during 24 h was decided. The patient evolved favorably; therefore, a few hours later, vasoactive drugs could be interrupted and subsequently extubation was carried out.

On the fourth day, she was discharged to the ward. The echocardiography on the fifth day revealed an LVEF improvement (30%). She went home on the ninth day and 3 months later a new echocardiography showed an LVEF of 50%.

PPCM is a rare cause of AHF in the last months of pregnancy or within 5 months after delivery, in the absence of demonstrable preexisting cardiac disease and identifiable cause, which determines cardiac failure, with echocardiographic evidence of left ventricular systolic dysfunction.[2,3]

There is no specific treatment for this disease. In general, the treatment of PPCM is similar to that of other forms of AHF. However, in the literature there are few cases that describe the satisfactory use of levosimendan in PPCM, with improved myocardial performance associated with hemodynamic and symptom improvement, and a recovery of left ventricular function.[2,4,5]

Levosimendan—calcium sensitizer—increases myocardial contractility without increasing oxygen requirements, and also induces peripheral and coronary vasodilation through the opening of ATP-sensitive potassium channels in vascular smooth muscle. Because levosimendan does not increase intracellular calcium levels, it is less likely than traditional inotropes to have deleterious effects on cardiac myocyte relaxation, cell survival, and arrhythmia induction.

In spite of its short half-life, effects are long-lasting due to the active metabolite OR-1896, which has an elimination half-life of 70–80 h in patients with heart failure.[2,5,6]

At present, although there exists literature about the beneficial effects of levosimendan in PPCM, it is also true that the reported cases are sparing and that the only treatment based on scientific evidence is the habitual used in AHF.
